# Extremophiles in a changing world

**DOI:** 10.1007/s00792-024-01341-7

**Published:** 2024-04-29

**Authors:** D. A. Cowan, S. V. Albers, G. Antranikian, H. Atomi, B. Averhoff, M. Basen, A. J. M. Driessen, M. Jebbar, Z. Kelman, M. Kerou, J. Littlechild, V. Müller, P. Schönheit, B. Siebers, K. Vorgias

**Affiliations:** 1 Centre for Microbial Ecology and Genomics, Department of Biochemistry, Genetics and Microbiology, University of Pretoria, Pretoria, 0002 South Africa; 2 Faculty of Biology, University of Freiburg, Freiburg, Germany; 3 Institute of Technical Biocatalysis, Hamburg University of Technology, 21073 Hamburg, Germany; 4 Graduate School of Engineering, Kyoto University, Kyoto, Japan; 5 Department of Molecular Microbiology and Bioenergetics, Institute of Molecular Biosciences, Goethe University Frankfurt, Frankfurt Am Main, Germany; 6 Department of Microbiology, Institute of Biological Sciences, University of Rostock, Rostock, Germany; 7 Groningen Biomolecular Sciences and Biotechnology Institute, University of Groningen, Nijenborgh 7, 9747 AG Groningen, The Netherlands; 8 Univ. Brest, CNRS, Ifremer, Laboratoire de Biologie Et d’Écologie Des Écosystèmes Marins Profonds (BEEP), IUEM, Rue Dumont d’Urville, 29280 Plouzané, France; 9 Institute for Bioscience and Biotechnology Research and the National Institute of Standards and Technology, Rockville, MD USA; 10 Department of Functional and Evolutionary Ecology, Faculty of Life Sciences, University of Vienna, Vienna, Austria; 11 Henry Wellcome Building for Biocatalysis, Faculty of Health and Life Sciences, University of Exeter, Exeter, UK; 12 Institute of General Microbiology, Christian Albrechts University, Kiel, Germany; 13 Molecular Enzyme Technology and Biochemistry (MEB), Environmental Microbiology and Biotechnology (EMB), Centre for Water and Environmental Research (CWE), University of Duisburg-Essen, 45117 Essen, Germany; 14 Biology Department and RI-Bio3, National and Kapodistrian University of Athens, Athens, Greece

**Keywords:** Bioeconomy, Bioenergy, Biomining, Bioproducts, Extremophiles, Gene discovery, Global Sustainability Goals, Metagenomics, Sustainable Development Goals, Thermophiles

## Abstract

Extremophiles and their products have been a major focus of research interest for over 40 years. Through this period, studies of these organisms have contributed hugely to many aspects of the fundamental and applied sciences, and to wider and more philosophical issues such as the origins of life and astrobiology. Our understanding of the cellular adaptations to extreme conditions (such as acid, temperature, pressure and more), of the mechanisms underpinning the stability of macromolecules, and of the subtleties, complexities and limits of fundamental biochemical processes has been informed by research on extremophiles. Extremophiles have also contributed numerous products and processes to the many fields of biotechnology, from diagnostics to bioremediation. Yet, after 40 years of dedicated research, there remains much to be discovered in this field. Fortunately, extremophiles remain an active and vibrant area of research. In the third decade of the twenty-first century, with decreasing global resources and a steadily increasing human population, the world’s attention has turned with increasing urgency to issues of sustainability. These global concerns were encapsulated and formalized by the United Nations with the adoption of the 2030 Agenda for Sustainable Development and the presentation of the seventeen Sustainable Development Goals (SDGs) in 2015. In the run-up to 2030, we consider the contributions that extremophiles have made, and will in the future make, to the SDGs.

## Introduction

Extremophiles and their products have contributed much to areas such as diagnostics and bioprocessing (Coker 2016). The most successful commercial examples of extremophilic products are the DNA manipulating enzymes (*Taq* polymerase, Vent^®^ polymerase), and numerous other extremophilic enzymes that play important roles in commercial drug biosynthesis, household products and large-scale biotransformations (Antranikian and Egorova 2007). Extremophilic organisms also continue to yield novel metabolic pathways, enzymes and control elements (e.g., Bräsen et al. 2014; Weisse et al. 2016; Saitsev et al. 2018; Johnsen et al. 2019, 2020, 2023; Stracke et al. 2020; Kuprat et al. 2021; Lewis et al. 2021).

In a world where research fields rise and fall, it is perhaps surprising that extremophile research remains a highly active and exciting topic. The continued interest in extremophile research has many causes. The concept that very high levels of evolutionary novelty, such as in the hyperthermophilic archaea, will be associated with molecular and functional novelty is borne out by recent metagenomic studies of hot pool and deep-sea hydrothermal vent microbiomes (Strazzulli et al. 2017; Iacono et al. 2020; Reichart et al. 2021) and metaviromes (Dávila-Ramos et al. 2019), and by the discovery of completely unique viruses in hydrothermal systems (Prangishvilli et al. 2017). Similarly, the notion that studies of organisms living at the outer edges of the ‘biological envelope’ can yield unique insights into the mechanisms of structural and functional adaptation and survival strategies has been supported by decades of research (see, for example, Gerday and Glansdorff 2007; Schmerling et al. 2022). Extremophiles remain one of the dominant themes in the field of astrobiology and the search for life on other planetary bodies (Thombre et al. 2020).

The global biotechnology industry has also retained an ongoing interest in extremophiles and their products (Krüger et al. 2018; Straub et al. 2018; Pfeiffer et al. 2021). The lesson of the past 30 years, since the heyday of Recombinant Biocatalysis Inc. and Diversa Corpn. (DeSantis et al. 2002), is that the road to commercial success is often driven top-down (from industry needs) rather than bottom-up (from scientific discoveries). Nonetheless, achieving true progress towards completely novel products and innovations requires a solid scientific basis—and often novel scientific discoveries that may be exploited, such as gene editing using the CRISPR Cas 9 system. CRISPR loci have been described in halophiles by Francisco Mojica (Mojica et al. 1993), who later proposed a role for these sequences in microbial immunity (Mojica et al. 2005). Proof of the continued interest in extremophiles as sources of academic and biotechnological novelty is evident in the fact that extremophile research is still well-funded (e.g.*,* in EU-programmes such as Horizon 2020).

It is necessary to add a caveat at this point. While extremophiles are already used in many fields of biotechnology and, as discussed below, hold great potential for future exploitation, these organisms are not without their practical limitations. The stringent optimal growth conditions for some extremophiles, and the costs associated with their growth at large (multi-kilolitre) scales, together impose significant challenges for industrial application.

However, the aim of this review is to look forward, not back; to speculate on the future of extremophiles in a changing world where issues of climate change, sustainability, constraints on energy, food systems, healthy soil and clean water supplies and global health challenges are all increasingly immediate and concerning. We also aim to highlight conceptual and practical gaps in our technical and knowledge base that currently inhibit our ability to fully exploit the resources available in the world of extremophiles (and other biological groups).

In less than 20 years since the commercialization of Next Generation nucleic acid sequencing platforms, the global research community is deluged in DNA sequence data. At the time of writing, many hundreds of publicly-available extremophilic metagenomic DNA sequence datasets, representing many Terabase-pairs of sequence data) are accessible in NCBI and other platforms. These datasets represent a resource of enormous scale: tens of thousands of complete (and incomplete) biosynthetic pathways and many millions of protein coding genes, a significant proportion of which (20–40%; e.g., Chen et al. 2021) cannot currently be annotated and are therefore of totally unknown function (Salzberg 2019). This sequence resource will continue to grow, possibly at an exponential rate. While this represents an ever-increasing resource, the gap between digitalized and experimental data will grow proportionally.

We note, as have others, that the rate of sequence data generation generally exceeds the rate of development of systems and processes capable of manipulating, interpreting, understanding and exploiting this sequence resource. The general issues of ‘data overload’ have been discussed in some detail elsewhere (Baker 2010).

## Gene discovery

The latest technologies available for the identification of genes (and/or gene products) in metagenomic DNA are broadly divided into functional metagenomic methods (most commonly function-based screening of metagenomic plasmid, phage, fosmid, cosmid or BAC/YAC expression libraries (Uchiyama and Miyazaki 2009; Iacono et al. 2022)), and gene-mining from assembled metagenome sequence data (Kenshole et al. 2021). Both approaches have substantial limitations.

In the former, expression screening is restricted to a very limited range of expression hosts, almost none of which are extremophiles (Uchiyama and Miyazaki 2009; Tripathi and Srivastava 2019). Issues such as the constraints of variable codon-usage, promoter sequence variations, the complexities of in vivo gene expression and domain-specific post-translational modification all limit the efficiency of expression screening of extremophile metagenomic DNA extracts. Even where screening is successful, scale-up of expression is largely constrained to a few highly engineered non-extremophilic expression hosts (Tripathi and Srivastava 2019). Expression screening is also limited by assay development: it is notable that two decades of gene mining metagenomic expression screening (often termed *functional metagenomics*) has largely exploited readily available chromogenic hydrolase substrates.

Some of the current gaps in the technology are obvious: ‘plug-and-play’ laboratory expression hosts for the major groups of extremophiles (e.g., thermophilic bacteria and archaea, psychrophiles, acidophiles, halophiles), where intracellular expression is highly impacted by the extracellular environment (temperature, pH, salt), are only available for a limited range of organisms. Probably the first thermophilic organism to be genetically manipulated was *Thermus thermophiles* (Oshima 1995), and this organism has now been engineered as a thermophilic host for the over-expression of proteins that cannot be expressed in mesophilic systems (De Rose et al. 2021a). However, the very high G + C content of the DNA of this organism generally means that genes to be expressed in this host system have to be synthesized.

The constant development of novel genetic tools for extremophilic archaea (Leigh et al. 2011; Atomi et al. 2012; Farkas et al. 2013; Pfeifer et al. 2021) and bacteria (Averhoff et al. 2021*)* is particularly promising. Examples include genetic methods to manipulate thermophiles *Pyrococcus furiosus* and *Thermococcus* spp. (Lipscomb et al. 2011; Sato et al. 2005; Thiel et al. 2014; Birien et al. 2018) and *Sulfolobus* (Wagner et al. 2012; Quehenberger et al. 2017) as well as halophilic (Peck et al. 2000; Bitan-Banin et al. 2003; Allers et al. 2004) and methanogenic (Susanti et al. 2019) archaea. Within the extremophilic bacteria, it is now possible to over-produce oxygen-sensitive proteins; e.g*.,* in the anaerobe *Thermoanaerobacter kivui* (Basen et al. 2018; Katsyv et al. 2021a), and a two-enzyme cascade from *Methanothermus fervidus* has been used successfully to produce the extremolyte cyclic di-phosphoglycerate, a small molecule that can stabilize proteins and DNA and has commercial application in the healthcare and cosmetic industries (de Rose et al. 2021b).

*Thermus thermophilus* is a particularly suitable candidate as a production platform for thermostable proteins, given its very high natural transformation frequencies (Cava et al. 2009; Averhoff et al. 2021). Six inducible promoters, the *P*_*arg*_, *P*_*dnaK*_, *P*_*scs-mdh*_* P*_*nar*_, *P*_*sip*_, and *P*_*pilA4*_ promoters, have been characterized and two systems for efficient protein over-production have been reported (Moreno et al 2003, 2005; Fujino et al. 2020; Kirchner and Averhof 2023). However, it has to be noted that the *P*_*nar*_ promoter is only induced under anaerobic conditions in the presence of nitrate and the *P*_*sip*_ is only induced by addition of 10 mM silica, which leads to growth inhibition (Moreno et al. 2005; Fujino et al. 2020).

It has been proposed that a viable alternative for the development of cell-based systems for the *in vitr*o overexpression and production of extremophilic proteins is the use of active learning in the optimization of the physico-chemical and biochemical process conditions (Borkowski et al. 2020).

While these developments provide some insights into the current and future potential of extremophilic protein production platforms, there remains considerable room for improvement. To the authors’ knowledge, no extremophilic organism has ever been engineered for very high-level (multi-g per L) protein production, a critical requirement for economic viability of any commercial commodity enzyme. However, an L-aminoacylase from *Thermococcus litoralis*, over-expressed in *Escherichia coli* or *Pseudomonas* systems at room temperature, is being used for large scale L-amino acid and amino acid analogue production at 50°C by Dr Reddys/Chirotech (Toogood et al. 2002). The concurrent use of a racemase enzyme capable of converting the unused D-enantiomer into the L-enantiomer gives full kinetic resolution (100% conversion) production.

## Metagenomic gene mining

The scope of gene mining using functional metagenomics is hugely constrained by the limited range of assay methods that can be readily adapted to high throughput screening (Markel et al. 2020). The development of new assay systems requires a strongly interdisciplinary approach; particularly the close collaboration between biochemists and synthetic organic chemists. Nevertheless, some very significant advances in metagenomic screening technology have been introduced over the past two decades. High throughput robotic liquid handling systems, coupled with advanced microfluidics technology (e.g., de Boer et al. 2005; Strutt et al. 2023) have made it possible to screen very large recombinant expression libraries in practical timescales.

It is argued that the future of ‘gene mining’ lies in silico: identification of novel genes in assembled metagenomic sequence datasets using bioinformatics tools (Koonin et al. 2021). The strengths of this approach are based around the scalability of the process, the speed and capacity of modern computational platforms and the increasing role of Artificial Intelligence (AI) systems (e.g., Saldívar-González et al. 2022). The release of AlphaFold, a Google DeepMind AI-based program, has dramatically increased the accuracy of protein structure prediction (Kryshtafovych et al. 2021), although the algorithm is only reliable for proteins that have related structures available in the protein PDB database. It has been reported that the two enzymes responsible for the biosynthesis of the extremolyte cyclic di-phosphoglycerate, 2PGK and cDPGS (for which no similar protein structures have been solved), give inaccurate AlphaFold predictions (De Rose et al. 2023).

**Fig. 1:**
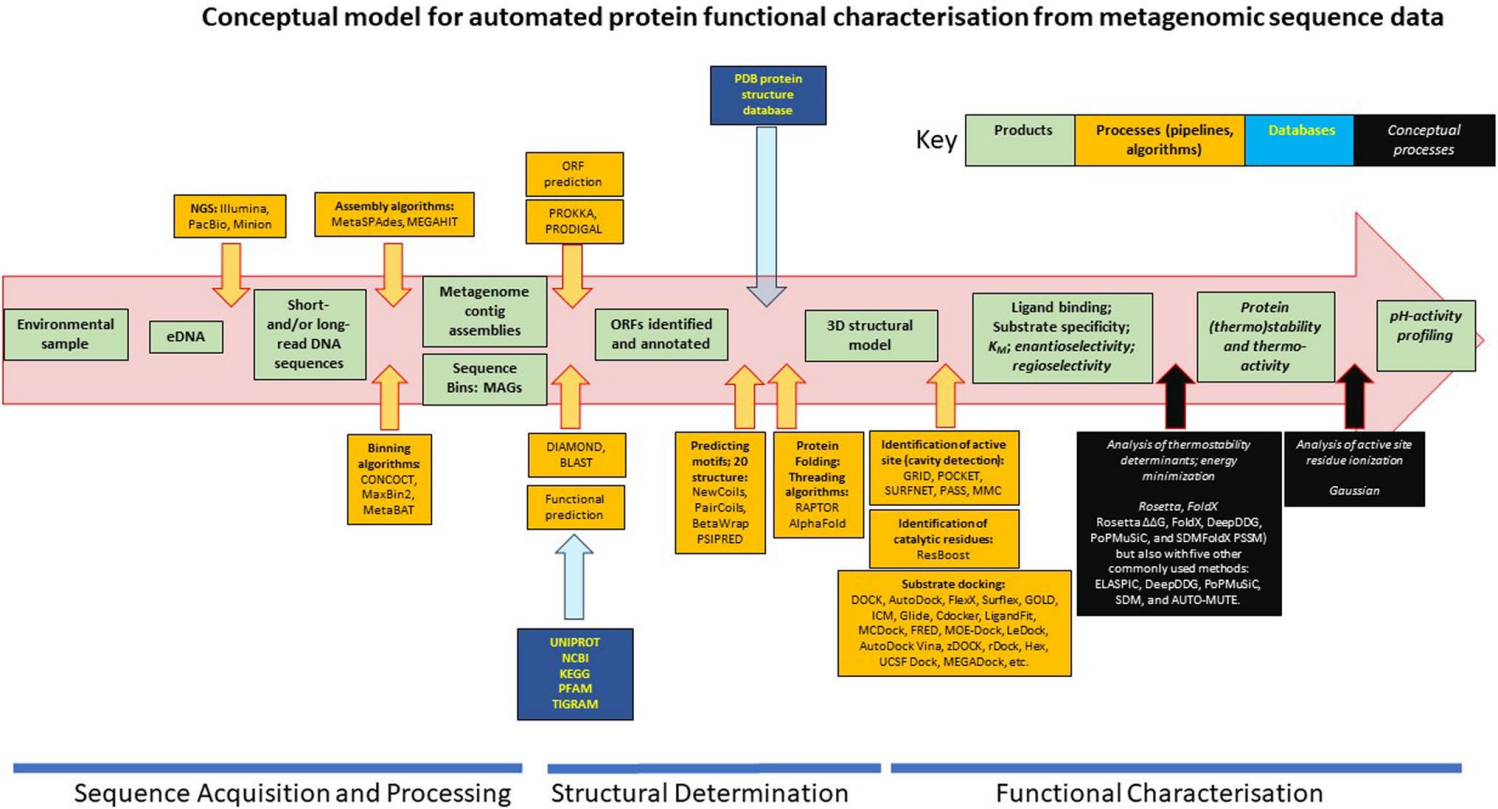
Conceptual model for automated protein functional characterisation from metagenomic sequence

A very wide array of bioinformatic tools is available for processing nucleic acid and protein sequence data along the pipeline from raw sequence to accurate prediction of a folded and functional protein (Fig. 1) (https://bio.tools/), and new algorithms and pipelines are released almost daily. Some, such as AlphaFold, have moved the field forward in quantum leaps (Jones and Thornton 2022). However, some fundamental gaps remain. Most of the tools for structural and functional prediction are homology-based, providing little useful information for the ‘dark sequence’ fraction of genomes, which can be as high as 50% in particularly novel extremophiles such as the Asgard archaea (MacLeod et al. 2019). Bioinformatic tools for prediction of subtle inter-molecular interactions, such as oligomerization, protein–ligand binding and enzyme–substrate docking generally lack sufficient resolution, and the computational capacity to quantitate some protein properties, such as enzyme catalytic rates as well as Intrinsically Disordered Regions (Necci et al. 2021), are not currently available. Furthermore, the many bioinformatic tools that do exist to process steps along the sequence-function pipeline (Fig. 1) are generally not coordinated or interlinked, and there are some fundamental discontinuities in data-exchange. For example, major protein functional databases such as BRENDA (https://www.brenda-enzymes.org/) are currently not coordinated with many processing algorithms, and new data on protein function may often not flow back into the sequence-function pipeline.

We freely acknowledge that the advances in science will resolve these issues in time, but argue that the development of coordinated bioinformatic systems and processes designed to accurately extract functional data on gene products from sequence data should be an immediate priority.

Nevertheless, there remains enormous scope (and need) for the development of new approaches to the discovery of new genes and processes, particularly those that come from the unknown (dark sequence) fraction of metagenomic data. Recent advances in correlative analysis of transcriptomes, proteomes and metabolomes in complex communities and enrichment cultures (i.e., meta-transcriptomics, meta-proteomics, meta-metabolomics) or in single cells open new horizons for bioprospecting. For example, the development of novel technologies such as the combination of (meta)genomics and activity-based protein profiling (ABPP: Cravatt et al. 2008; Fang et al. 2021) makes it possible to target specific active enzyme classes in vivo, for subsequent quantification and identification by downstream LC–MS/MS. This approach has been successfully used for the identification of extremozymes in pure cultures (e.g., *Sulfolobus* spp*.*, *Haloferax volcanii* and *Thermococcus* sp.: Zweerink et al. 2017) as well as for the direct profiling of extremophilic microbial community samples in their native environment (environmental ABPP, eABPP: Ninck et al. 2022).

## Extremophiles and the sustainable development goals

**Fig. 2:**
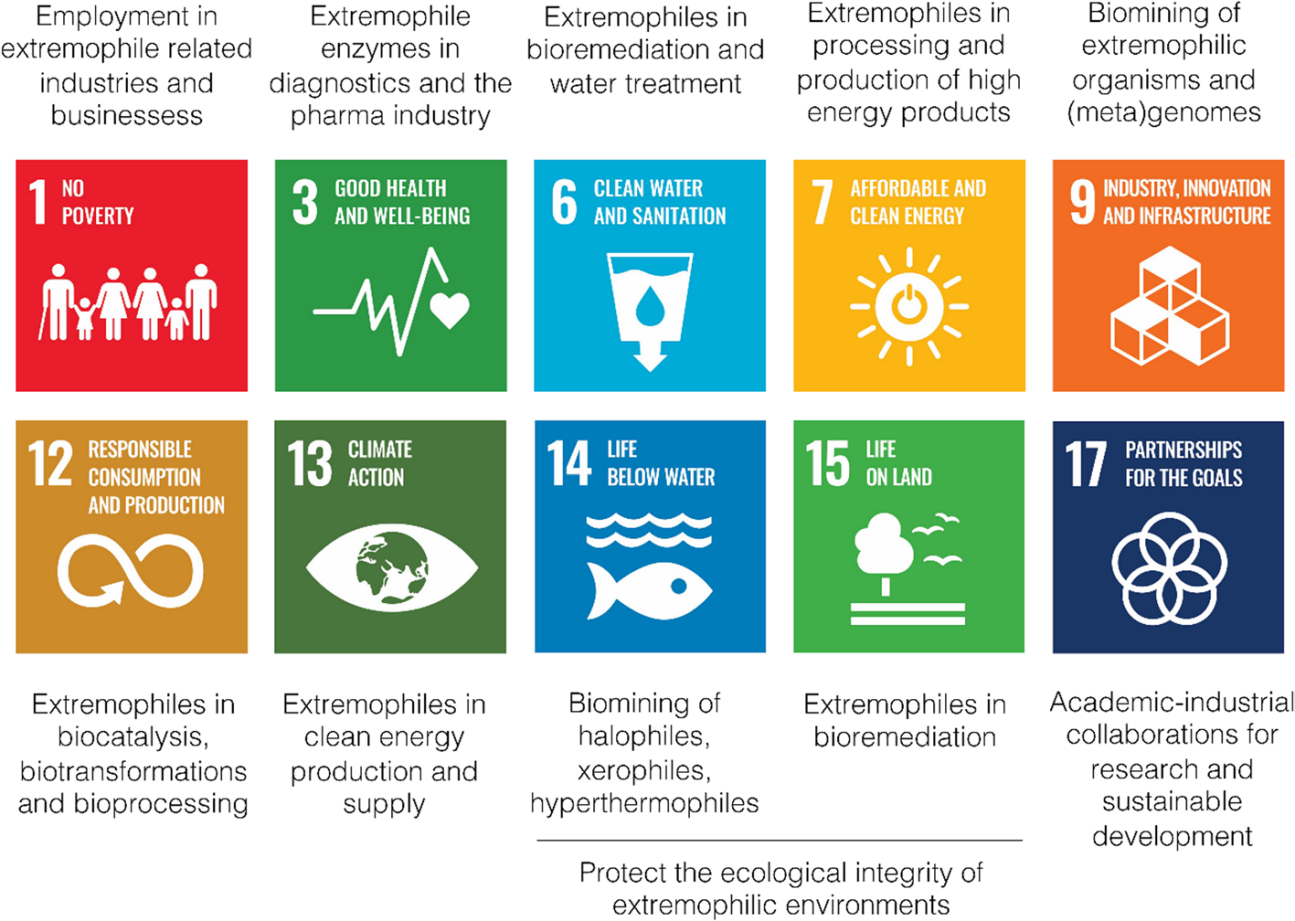
Contributions of extremophiles to the UN Sustainable Development Goals

In a changing world, the United Nations has defined a list of goals that aim to transform our world. (17 sustainable development goals; SDG). The SDGs are a call to action to end poverty and inequality, ensure the sustainability of the processes underlying our societies and economies, and protect the biosphere as the foundation of these objectives. We postulate that basic and applied research in extremophiles can strongly contribute to achieving these goals. In this review, we focus on specific SDGs where we believe extremophiles have made and will continue to make a significant contribution (Fig. 2).

### SDG3: good health and well-being

This SDG incorporates many elements relating to communicable and non-communicable human diseases, for most of which extremophilic organisms and their products have no obvious contribution. However, several extremophilic enzymes have played critically important roles in diagnostics and disease research. The most obvious and well-known is *Taq* DNA polymerase, originally sourced from the extreme thermophile *Thermus aquaticus* (Innis et al. 1988). This enzyme has a projected market value in 2023 of around $US0.35 billion (https://www.futuremarketinsights.com/reports/dna-polymerase-market). The DNA polymerase market is dominated by *Taq polymerase* and various engineered variants, but with contributions from other hyperthermophilic functional homologues. Interestingly, the process (PCR) and its core enzyme, which until recently were known only in the research and diagnostic fields, have now become a matter of public knowledge, thanks to the globally important process of Covid-19 PCR testing.

Haloarchaeal carotenoids, in particular bacterioruberin, are especially exciting as they might represent the first case of extremolytes with a demonstrated therapeutic effect in vitro. Bacterioruberin, a rare C_50_ carotenoid synthesized by most haloarchaea has been shown to exhibit the highest antioxidant capacity of all known carotenoids, including ﻿ß-carotene (Mandelli et al. 2012), and were recently shown in in vitro studies to have a cytotoxic effect on human breast cancer lines (Giani et al. 2021, 2023). A novel C_50_ carotenoid were also recently identified from *Natrialba* sp. M6, and was also shown to exert significantly higher anticancer effects than standard anticancer drugs, as well the ability to suppress the replication of the hepatitis viruses HCV and HBV (Hegazy et al. 2020).

**Fig. 3:**
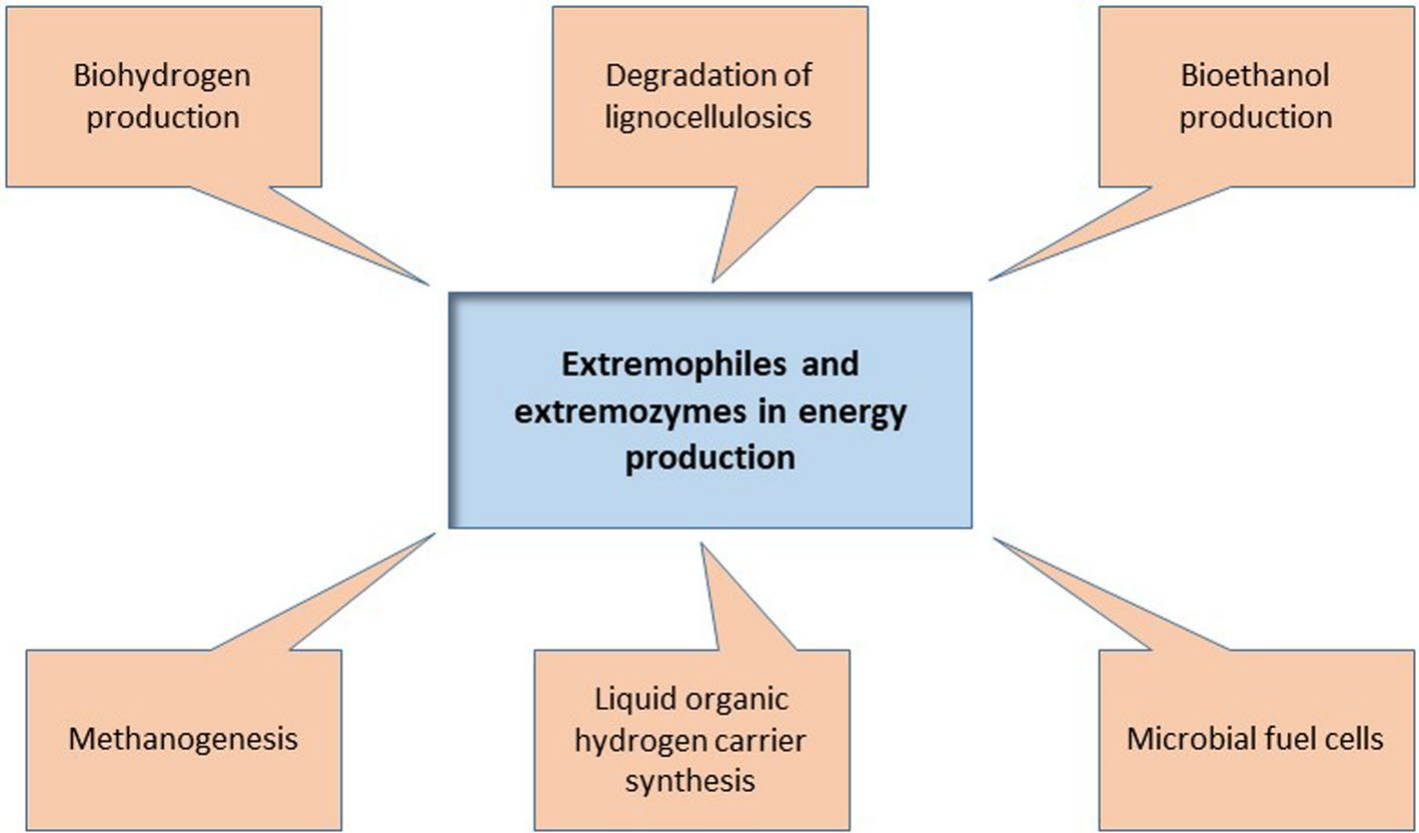
Extremophiles and SDG7 (Affordable and Clean Energy)

**SDG7: Affordable and clean energy** is goal number 7 as defined by the United Nations, and arguably the one where extremophiles may make the greatest contribution (summarized in Fig. 3). Currently, the world’s economy is based on the combustion of fossil fuels but concerns about anthropogenic climate change and global warming have led to many initiatives, including those exploiting extremophiles, to replace fossil-fuel based energy carriers. The biological production of fuels from different feedstocks has been experimentally addressed for some decades; early studies focused on sugars as feedstock but this approach competes with human nutrition (the food *versus* fuel discussion (Naik et al. 2010)). Therefore, sustainable production of fuels and other products from biomass by fermentation should rely on plant-based feedstocks that are not used for human nutrition, such as wood and its constituents (lignocellulose). The use of thermophilic enzymes for degradation of lignocellulosic substrates to simple sugars, for subsequent fermentation to generate value-added products, has been a focus of research and development for more than three decades (e.g., Blumer-Schuette et al. 2014; Botha et al. 2018; Singh et al. 2021).

Numerous thermophilic bacteria and fungi are known to degrade cellulose and lignocellulose, yielding ethanol and hydrogen (Andlar et al. 2018; Chukwuma et al. 2021). High temperatures facilitate the degradation of the complex feedstocks and, indeed, growth rates on crystalline cellulose in thermophiles are generally superior to those of mesophiles (Blumer-Schuette et al. 2015), one of the reasons that the US DOE-funded BioEnergy Science Center has focused on biomass conversion in two model thermophilic lignocellulolytic bacteria, *Clostridium thermocellum* and *Caldicellulosiruptor bescii* (Gilna et al. 2017). Both bacteria have been shown to be highly cellulolytic, efficiently degrading high loads of unpretreated lignocellulose (Basen et al. 2014a; Straub et al. 2019; Kubis et al. 2022), but they differ in their mechanism of plant biomass deconstruction. While *C. thermocellum* harbors the outer cell wall-attached cellulosome, a huge multienzyme apparatus harboring Carbohydrate Active enZymes (CAZymes) such as glycosyl hydrolases and pectate lyases (Artzi et al. 2017), *Caldicellulosiruptor* sp. mostly use secreted multidomain CAZymes for biomass deconstruction (Yang et al. 2009; Kataeva et al. 2013) next to surface-attached glycosyl hydrolases (Conway et al. 2016). Both organisms harbor arsenals of thermostable CAZymes for putative industrial applications. As an example, the multidomain endo/exo-cellulase CelA from *Caldicellulosiruptor bescii* outperformed the commercial (fungal) cellulase blend CTec2 on crystalline cellulose (Brunecky et al. 2017). *Caldicellulosiruptor* species are known to achieve high hydrogen yields from sugars, close to the maximum of 4 mols H_2_/mol C6 unit (the Thauer limit) (Van de Werken et al. 2008). This yield may even be improved in the future by co-culture experiments that oxidize the acetate/ethanol produced by fermentation to H_2_ + CO_2_. Thermodynamically, this is challenging, but recent advances in understanding production of molecular hydrogen from high potential electrons by the process called electron bifurcation (Müller et al. 2018; Peters et al. 2016; Buckel and Thauer 2018) will lead to further improvement.

Production of fuels such as ethanol from biomass or biomass-derived sugars by (hyper)thermophiles has been studied extensively and is still an area of very productive research (Olson et al. 2015). Genetic tools have been established for several cellulolytic microbes (C. *bescii* and *C. thermocellum*: Guss et al. 2012; Chung et al. 2013; Lipscomb et al. 2016), and used to improve ethanol yields in both organisms (Chung et al. 2014; Argyros et al. 2011; Olson et al. 2015 and references therein). However, this remains challenging as a potentially commercial process as they are not native ethanol producers (*C. bescii*) and produce ethanol as one of many products (*C. thermocellum*). Conversely, the related thermophilic genera, *Thermoanaerobacterium* and *Thermoanaerobacter*, naturally produce ethanol at comparably high yields (Wiegel et al. 1981), which have been further improved by deleting the production of other fermentation products such as lactate and acetate (Argyros et al. 2011; Olson et al. 2015). The understanding of ethanol production in these species may serve as a template for improvement of ethanol production in *C. thermocellum* or *C. bescii* or in other thermophilic anaerobes. One key enzyme may be the bifunctional aldehyde/alcohol dehydrogenase AdhE (Lo et al. 2015; Hitscher et al. 2021), which is essential for ethanol production from acetyl-Coenzyme A in some fermentative organisms. Fermentative microbes, however, preferentially produce acetate from acetyl-CoA as a means of generating additional ATP: this represents an energetic barrier to ethanol production in such organisms. Moreover, reduced ferredoxin (Fd_red_) is often produced during pyruvate oxidation, and Fd_red_ is not utilized by AdhE. A viable alternative is the use of a membrane-bound enzyme complex that oxidizes Fd_red_ and reduces NAD^+^ at the same time; the energy-conserving Rnf-complex (Biegel et al. 2010). The use of genetic engineering to overproduce this complex may be a route to higher ethanol yields (Williams-Rhaesa et al. 2018). Other thermophiles contain an enzyme, aldehyde-ferredoxin oxidoreductase (AOR), which reduces acetate to acetaldehyde (Mukund and Adams 1991), where acetate may then be converted to ethanol by an alcohol dehydrogenase. Some metabolic engineering of AOR-containing pathways has already been performed: for example, in the hyperthermophilic archaeon *P. furiosus* (Basen et al. 2014b) and in *Caldicellulosiruptor* sp. (Rubinstein et al. 2020). AOR not only oxidizes acetate but also higher carbon chain acids when provided to the bacteria in the medium (Hitschler et al. 2021; Nissen and Basen 2019); thus, fuels with higher energy density such as butanol or even hexanol can be produced. It is possible that this conceptual process will become a reality in the near future, using extremophiles in the production of long chain alcohols (kerosene). Recently, production of ethanol was achieved at 95°C by an engineered pathway in *P. furiosus*, using CO as a substrate (Lipscomb et al. 2023).

Biofuel production from biomass is always accompanied by the production of carbon dioxide, and although there is no net production of carbon dioxide in the process it is desirable to limit further increases in atmospheric CO_2_ levels. CO_2_ produced by processes under high temperatures can be removed by hot acetogenesis. Acetogens such as *Moorella thermoacetica* or *Thermoanaerobacter kivui* fix CO_2_ by the Wood-Ljungdahl pathway to acetyl-CoA that is further reduced to acetate (Basen and Müller 2017; Rosenbaum and Müller 2023). Electrons for the reduction of CO_2_ can be derived from oxidation of molecular hydrogen, from methanol (or other methyl-group containing substrates) or carbon monoxide. Even electrons derived from an anode can be used (electrosynthesis). Although the major pathways, the enzymes and genes involved are known (Katsyv et al. 2021b), there are still gaps: for example, what is the role of the two different energy-converting hydrogenases (Schölmerich and Müller 2019) and are there more, cytochrome-containing, energy conservation mechanisms (Rosenbaum and Müller 2021)? The latter is of special importance since acetogens are energy limited and can only produce low energy products such as acetate or some ethanol. Higher carbon chain compounds are only produced by a few species and only on very minor amounts. Metabolic engineering of (thermophilic) acetogens is well on its way but production of value-added compounds is restricted thermodynamically. One of the challenges of the future is to overcome these energetic barriers (Katsyv and Müller 2020).

CO_2_ can also be reduced to methane by methanogenesis, in a pathway similar to acetogenesis, carried out by thermophilic methanogens (Thauer et al. 2008). Electrons can be derived from molecular hydrogen but also electrochemically and the company Elektrochaea (https://www.electrochaea.com) is leading the technological development of commercial biomethanization, or CO_2_-based biological CH_4_ production (CO_2_-BMP), a process with potential as chemical energy storage of excess electricity (Bernacchi and Herwig 2016; Bernacchi et al. 2016). Recent physiological screening of 80 cultivated methanogenic archaea yielded a number of high-performance strains for future development of high temperature biomethanization (Mauerhofer et al. 2021). Biomethanation of carbon monoxide has also been achieved by using a synthetic hyperthermophilic archaeal consortia (Zipperle et al. 2021).

Thermophilic bacteria such as the anaerobe *Thermovibrio ammonificans*, which contain a highly thermostable α-carbonic anhydrase, are being developed for CO_2_ capture (James et al. 2014). This enzyme has been immobilized in industrial-scale bioreactors to remove CO_2_ from the environment (CO_2_ solutions, Quebec, Canada; www.co2solutions.com).

Molecular hydrogen has been considered as an attractive, alternative energy carrier (Rosen and Koohi-Fayegh 2016), as has ‘hythane’, a mixture of hydrogen and methane (Ghosh and Kar 2022). Hydrogen can be produced in various ways. If generated by water splitting using renewable energy sources (i.e., solar-, wind-, hydro- or geothermal-power) there is no net CO_2_ production in the production process. However, such processes are not yet operating at a scale large enough to impact fossil fuel usage. High temperature processing may improve production efficiencies. The thermophilic archaeon *Thermococcus onnurineus* and the thermophilic bacterium *Thermoanaerobacter kivui* have the highest-ever reported rates of hydrogen production from formic acid (Müller 2019; Burger et al. 2022). While the former uses a membrane-bound enzyme for formate oxidation (Kim et al. 2010; Bae et al. 2015), the latter has a soluble enzyme: hydrogen-dependent CO_2_ reductase (Schuchmann and Müller 2015; Dietrich et al. 2022). A current limitation is the low cell density these organisms achieve in bioreactors, offering considerable scope for future improvement. An interesting aspect is that carbon monoxide, a component of synthesis gas (a waste product from steel milling) can be oxidized to molecular hydrogen (and CO_2_) at high temperatures (Kim et al. 2014; Weghoff and Müller 2016).

One of the biggest challenges in the hydrogen industry is storage and transport of this highly explosive gas. One possibility is to bind molecular hydrogen to create a liquid, organic hydrogen carrier (LOHC), such as formate. Formate is produced from H_2_ + CO_2_ by chemical catalysis but also by the hydrogen-dependent CO_2_ reductase (HDCR; Müller 2019). The HDCR from the thermophile *T. kivui* has the highest-ever reported rates for CO_2_ reduction with molecular hydrogen to formate (Schwarz et al. 2018; Schwarz and Müller 2020). LOHC formate can be shipped over long distances and reoxidized at the target location to hydrogen (and CO_2_). Recently, a biobattery has been developed in which cells produce formate from hydrogen and carbon dioxide during the day and then reverse the reaction at night to generate molecular hydrogen (Schwartz et al. 2022).

### SDG9: industry, innovation and infrastructure

#### Establishment of advanced extremophilic protein expression and metabolic engineering platform

Although extremophiles and their extremozymes continue to attract attention for commercial and industrial applications, some taxa, particularly extremophilic archaea, are still under-exploited compared to their mesophilic counterparts. The different lifestyles of extremophiles, with their molecular and physiological adaptations to the biotic limits of temperature, pH and salt, often match the harsh process conditions required for industrial processes such as lignocellulosic biomass conversion. However, the ability to engineer most extremophiles for industrial use remains limited. For a limited number of model extremophilic organisms such as *Sulfolobus acidocaldarius, Saccharolobus solfataricus, Pyrococcus furiosus, Thermococcus kodakarensis, Thermus thermophilus, Haloferax volcanii, Halobacterium salinarum, Methanocaldococcus jannaschii* and *Methanothermobacter thermautotrophicus,* advanced genetic systems have been developed to facilitate protein expression, gene deletion and metabolic engineering (e.g., Schocke et al. 2019). However, one major challenge for future commercial applications is the need to generate very high cell densities in large-scale bioreactors. Some advances have been reported: for example, process engineering developments for *S. acidocaldarius* culture medium optimization have yielding up to 35 g dry cell weight per litre fermentation broth (OD_650_ 50–60: Quehenberger et al. 2019). This compares well with wet cell yields of > 100 g.L^−1^ for some bacterial and yeast fermentations (Shay et al. 1987) although industrial fermentations achieve very much higher biomass yields.

*Sulfolobus* cell biomass has been used for production of archaeal liposomes (archaeosomes), which can serve as delivery systems for drugs such as vaccines, proteins, peptides and nucleic acids or for trehalose production (Rastädter et al. 2020). However, further process engineering developments are critical prerequisites to establishing extremophiles as metabolic engineering platforms and cell factories.

### SDG12: responsible consumption and production

Currently, the world’s fossil-fuel based economy is essentially linear, where the production of raw materials, the manufacturing of goods and their subsequent consumption patterns are frequently unsustainable, energy-inefficient processes which produce high volumes of waste and greenhouse gasses. For the transition to a circular economy, there is an urgent requirement for novel products that can be readily recycled back into the value chain or degraded without harmful effects for the environment.

Haloarchaea are a particularly interesting group of extremophiles that produce a rich repertoire of products with potential or actual uses in medicine (e.g., haloarchaeal bacteriorhodopsins in bioelectronics: reviewed by Pfeifer et al. 2021), or in material production (e.g., polyhydroxyalkanoates (PHA)). PHAs are a group of diverse bio-polyesters produced by microorganisms as carbon polyester storage compounds, and can be developed as biodegradable and sustainably sourced alternatives to fossil fuel-based plastics. Alongside the widely exploited mesophilic bacterial producers, haloarchaea have been developed as tools for PHA production, as they offer certain advantages over bacterial producers (Dietrich et al. 2017; Pfeifer et al. 2021). In an effort to connect CO_2_ fixation (and thereby removal) to production of added-value compounds *Methanococcus maripaludis,* a mesophilic autotrophic methanogenic archaeon, was recently engineered to produce PHAs by diverting the flow of acetyl-CoA to various biosynthetic pathways, paving the way for similar attempts with extremophiles (Thevasundaram et al. 2022). While not yet developed to industrial scale, this technology is considered to have considerable future promise in replacing fossil fuel-based plastics.

The establishment of safe and sustainable food systems has emerged as one of the most important focus points in the international agenda (e.g. Horizon Europe Cluster 6, SDGs 2, 12, 13). In the search for sustainable alternative protein sources suitable for human and animal diets, microbial protein is emerging as one of many promising alternatives (Matassa et al. 2016; Bajic et al. 2023).

Autotrophic, hydrogenotrophic thermophilic methanogens excrete all 20 proteinogenic amino acids into culture media, with the amino acid mixture composition varying depending on growth conditions (Taubner et al. 2023). The physiological reason for this remains unexplored, but the trait has been observed in syntrophic interactions involving methanogens in various ecosystems, and is therefore probably of ecophysiological significance (e.g. Imachi et al. 2020). A recently established startup company, Arkeon GmbH (https://arkeon.bio/), is developing a one-step fermentation process to upscale the production of amino acids from the genus *Methanothermobacter* as a sustainable protein source for the food industry (Turrell 2023). The added value of this technology is that the process can use CO_2_ from industrial sources, while the CH_4_ output could be used as a biofuel.

Another example with relevance to SDG12 (and SDG3) would be in the use of thermophilic sugar isomerases for the synthesis of new nutraceuticals (De Rose et al. 2021a), as sugar-free products which could combat chronic ‘lifestyle’ diseases such as diabetes, obesity, hyperlipidemia, and hypertension (all linked to high intakes of sugar and fatty foods). A thermostable D-Lyxose isomerase enzyme from a hyperthermophilic *Thermofilum* sp. shows activity up to 95°C. This enzyme is capable of producing D-mannose and L-ribose, both of which have applications in the food, cosmetic and pharmaceutical industries.

Extremophilic enzymes, the robust structures of which are well suited to the demands of synthetic industrial processes, play important roles in sustainable chemical synthesis. In particular, the unique stereoselectivity and enantiospecificity of some of these enzymes finds application in the synthesis of new drug intermediates (Littlechild 2015a, b). Enzymes that have evolved under different evolutionary pressures often show novel structural features, changes in active site tunnels, increased substrate promiscuity or different substrate stereo-specificities from related homologues that have previously been identified. Sequence-based screening of thermophilic metagenomes (Wohlgemuth et al. 2018) has yielded novel limonene and α/β class epoxide hydrolases; enzymes of special interest to the pharmaceutical industries (Ferrandi, et al. 2015, 2018).

The aminotransferases, which are capable of introducing chiral amines, are another important group of enzymes for the production of pharmaceuticals. A range of stereoselective aminotransferases, from the thermophilic archaea *Sulfolobus sulfotaricus* (Sayer et al. 2012), *Geoglobus acetivorans* and *Archaeoglobus fulgidus* (Isupov et al. 2019), have been developed as part of the enzyme toolkit for simplifying chemical synthesis pathways. For example, recombinant gluconate dehydratase from the hyperthermophilic crenarchaeon *Thermoproteus tenax* has been used for the production of chiral 2-keto-3 deoxy gluconate (KDG: Matsubara et al. 2014). The resource-efficient catalyst preparation (two precipitations), in combination with a one-step biocatalytic process, substitutes for a ten-step chemical synthesis and results in stereochemically pure KDG without side-product formation (90% yield).

It is now accepted that using enzymes in chemical synthesis is an economically viable route for producing a variety of new pharmaceuticals (Alcántara et al. 2022) and extremophile enzymes will play an important role in this achievement.

### SDGs 14, 15: life below water, life on land

These goals address the conservation, restoration and sustainable use of marine and terrestrial ecosystems. Currently, a significant proportion of global marine, freshwater and terrestrial ecosystems are polluted by industrial and urban wastes (e.g., petroleum, mining waste, agricultural runoff and hazardous chemicals). Bioremediation is a particularly attractive option for reducing the negative impacts of pollution, due to its sustainable and cost-effectiveness nature. The tolerance of extremophiles to environmental extremes (including high concentrations of heavy metals or xenobiotics) makes them particularly suited for some bioremediation processes. A few examples of advances in this area of research are discussed here, but for thorough reviews we direct the readers elsewhere (Krzmarzick et al. 2018; Kaushik et al. 2021).

Restoration of sites contaminated with crude oil or petroleum necessitates the degradation of a variety of aliphatic, branched and aromatic hydrocarbon compounds as well as other organic compounds. Pathways for activation and degradation have been found among the members of *Halobacteriota*, *Sulfolobales*, while methanogenic archaea often perform the final part of the process (Shukla and Singh 2020; Park and Park 2018). Excitingly, novel lineages performing anaerobic oxidation of alkanes are continuously being discovered among bacteria and archaea (termed ANME, anaerobic methane-oxidizing archaea, and ANKA, anaerobic multicarbon alkane-degrading archaea), with the latter using phylogenetically and functionally divergent variants of the methyl-coenzyme M reductase (MCR), the key enzyme of methanogenesis operating in reverse (Wegener et al. 2022).

Extremophiles encode multiple survival strategies against heavy metal toxicity in their environment, which can be harnessed for bioremediation of heavy metal and radionuclide contaminated sites (Marques 2018; Krzmarzick et al. 2018; Kaushik et al. 2021). These include bioadsorption to their cell surface or extracellular polymeric substances produced by them and biotransformation through enzymatic oxidation or reduction and subsequent precipitation or accumulation of organic or mineral metal complexes. Most of these strategies are found in thermophilic and acidophilic archaea and bacteria, and in Haloarchaea. Genetically tractable organisms such as members of the genus *Deinococcus* have successfully been engineered for enhanced radiation and heavy metal resistance and detoxification (Brim et al. 2000, 2003). As an alternative strategy, native microbial communities in contaminated sites have also been harnessed for natural bioremediation, assisted by biostimulation or bioaugmentation. This has been extensively researched in the case of acid mine drainage (Rambabu et al. 2020).

Extremophiles have also the potential to play a part in addressing the major driver of climate change; rising atmospheric CO_2_ concentrations. Natural mineralization of CO_2_ into mineral carbonates is a very slow process, where the rate limiting step is the hydration of dissolved CO_2_ to form HCO_3_^−^ (bicarbonate). This reaction can be performed biologically by carbonic anhydrases (reviewed in Bose and Satyanarayana 2017; de Oliveira Maciel et al. 2022; Steger et al. 2022), widespread bidirectional enzymes that maintain the carbonate equilibrium in both prokaryotes and eukaryotes, a crucial function for a number of physiological processes such as pH maintenance, ion transport and respiration. Biomineralization of CO_2_ at industrial combustion sites necessitates the use of thermo-alkali-stable carbonic anhydrases, as alkaline pH is needed for the reaction balance to shift towards bicarbonate. A number of enzymes with the desired properties have been characterized from archaea (*Sulfurihydrogenibium azorense*, *S. yellowstonense* YO3AOP1; De Luca et al. 2015) and bacteria (*Bacillus halodurans)*, while metagenomics has revealed the presence of novel carbonic anhydrase variants in eDNA (environmental DNA) sequence data from extremophilic environments (reviewed in Steger et al. 2022)*.* Lab-scale and pilot studies using these thermo-alkali-stable carbonic anhydrases have been shown to be effective in sequestering CO_2_ from flue gas (Capasso et al. 2012; Faridi and Satyanarayana 2016). While industrial scale processes are still in development, the construction of a CO_2_ capture facility utilizing CA was announced by the companies Saipem and Novozymes in 2021 (https://www.novozymes.com/en/news/novozymes-deliver-strategy-carbon-capture-collaboration-agreement).

## Extremophiles as models for life on other planets

Studying extremophiles can provide valuable insights into the potential for life on other planets, as these organisms demonstrate the capacity to thrive in environments that were once considered inhospitable (Cavicchioli 2002). By studying how such organisms survive and reproduce in extreme environments, we can better define the boundaries of habitability and refine our criteria for identifying potential habitable zones on other planets. Further, extremophiles can help us identify potential biosignatures—signs of past or present life—on other planets. By understanding the types of molecules and metabolic processes that extremophiles use to survive, we can search for similar signatures in the atmospheres or surface materials of other planets. Hence, extremophiles provide a basis for astrobiologists to develop models and theories on the potential types of life that could exist on other planets.

As an example, the exposure of extremophiles to simulated extreme conditions of other planets (such as Martian temperatures or the high-pressure environments of some of Jupiter’s moons), can yield insights into whether similar organisms could potentially survive there (e.g. Taubner et al. 2015, 2018; Favreau et al. 2023). Mars, with its cold arid conditions but a history of surface liquid water, is a prime target for the search for evidence of past life or even extant life. The hypothetical existence of extremophiles on other planetary bodies and systems (e.g., the Venusian atmosphere, the vapour plumes of the Saturnian moon Enceladus, and the icy depths of Europa) has been discussed in some detail (e.g., Seckbach and Stan-Lotter 2020; Schultz et al. 2023).

Studies of extremophiles contribute to our understanding of the limits of life’s resilience. This knowledge is important in preventing contamination of other planets with Earth microbes and will provide a valid basis of the design of future planetary protection protocols.

Extremophile research can contribute to our understanding of the origins of life. The extreme environments of modern Earth may resemble the conditions on early Earth over the period when life is believed to have originated. By studying how these organisms survive in such conditions, we can infer how life might have emerged and evolved in the early stages of our planet’s history. In addition, extremophiles may provide clues to the synthesis of important molecules and structures such as nucleic acids, protein-based catalysts and lipid membranes, and provide clues for prebiotic chemistry.

Hydrothermal vents on the ocean floor are extreme environments where life thrives in the complete absence of sunlight, relying completely on chemosynthesis for energy acquisition. One of the emerging theories is that these environments are analogous to the conditions that might have existed during the early evolution of life. Further, the burst of metagenomic studies of extreme environments has refined our understanding of the genetic makeup and evolutionary relationships of organisms, as well as eukaryogenesis, the process that led to the creation of the first eukaryotic cells. Extremophiles are often found to be deeply rooted in the tree of life, whereas in recent years new clades of archaea were discovered that are rooted in the eukaryotic tree of life lending further support for the symbiogenesis theory for the creation of eukaryotic cells.

## Conclusions

Over the past 50 years, extremophiles have contributed to many fields of biotechnology, spanning the full spectrum from basic research to large-scale industrial applications. The unique properties of these organisms and their constituents, driven by evolutionary processes and the properties of their environments, are frequently found to be well matched with the stringent requirements of the process or system in which they are used.

The future applications of extremophiles and extremophilic products, particularly in addressing the critical needs of the SDGs, will come, as they have in the past, from expanding the extremophilic ‘toolbox’ (Antranikian and Streit 2022) and from precise matching the properties of extremophilic ‘tools’ with process requirements (as expressed in the concept of the *Ideal Biocatalyst*: Burton et al. 2002).

We argue most strongly for the need for a continued emphasis on fundamental research in the field of extremophiles, since new tools to address the current and future challenges of society, industry and the environment inevitably come from fundamental understanding of the diversity, structure, function and properties of extremophiles and their constituents.

We also argue for a need to value the fundamental resources from which extremophiles are acquired; most especially a need to secure and protect specialized and/or limited extremophilic environments which are under threat from destructive commercial exploitation (e.g., mining Li-rich hypersaline lakes for the electronic industry, and tapping hydrothermal sources for energy production). Such habitats contain substantially untapped genetic resources (e.g., Antranikian et al. 2017) which, with future developments in computational power and sophistication (Kruger et al. 2020), will be increasingly accessible in the search for usable and valuable bioproducts.
